# BDS-3 Broadcast Ephemeris Orbit Correction Model Based on Improved PSO Combined with BP Neural Network

**DOI:** 10.1155/2022/4027667

**Published:** 2022-09-26

**Authors:** Jiebo Peng, Feng Liu, Wenjin Hu

**Affiliations:** School of Computer Science, Xi'an Polytechnic University, Xi'an, Shaanxi, China

## Abstract

During the operation of navigation satellites, errors in the broadcast ephemeris orbits are caused by the influence of ingress factors and other factors. To address this phenomenon, this paper examines the use of the computational intelligence (CI) methods to implement track correction and to develop an optimized BP neural network model based on an improved particle swarm algorithm. The model improves the inertia weights and learning factor parameters of the particle swarm optimization (PSO) algorithm to improve the global optimization capability and accelerate the convergence speed. The improved PSO (IPSO) algorithm is used to perform a global optimization search for the hyperparameters of the BP neural network, and then the neural network model is trained by broadcast ephemeris Keplerian root number and regression parameters. The model is validated using the broadcast ephemeris data of the BDS-3 MEO and IGSO satellites, and the mean square error correction rate of multiple satellites with different correction models shows that the error correction rate of the IPSO-BPNN model can reach 70.2–84% and the error correction rate can be improved by 14.2–56.8% compared with the PSO-BPNN model. The proposed algorithm provides an important reference for BDS-3 and other global navigation satellite systems for improving the accuracy of satellite orbit determination.

## 1. Introduction

The BeiDou satellite navigation system is a global satellite navigation system built and operated independently by China. In 2020, the third phase of the BeiDou system (BDS-3) was completed and it is now capable of providing high precision positioning and timing services to the world. The accuracy of the satellite ephemeris, which includes the broadcast ephemeris and postevent precision ephemeris, is directly tied to the accuracy of satellite positioning. The broadcast ephemeris has the feature of real time, but the accuracy is not sufficiently high. The precision ephemeris can be approximated as the true value of satellite coordinates with high accuracy, but the data have a high delay and cannot provide navigation and positioning services. Ensuring the accuracy of broadcast ephemeris has been a challenge that has been studied by many researchers [1–3]. The parameters of the broadcast ephemeris forecast can be optimized by combining mathematical and dynamical models to improve the positioning and timing accuracy. The uncertainty of the broadcast ephemeris spatial regimes and the high rate of change of the parameters make it difficult to improve the broadcast ephemeris accuracy by optimizing the dynamical model. In the process of analyzing the broadcast ephemeris orbit error data, it has been found that a new breakthrough approach can be identified from the error data itself by correlating the broadcast ephemeris parameters with the error data to reduce the broadcast ephemeris error and improve the accuracy of the broadcast ephemeris orbit [4, 5].

Broadcast ephemeris errors are time series type data, and numerous scholars have contributed to the prediction of time series data with good prediction results, which include the use of deep learning methods and neural network models. Rui Yan et al. compared the prediction performance in air quality prediction using BPNN, CNN, LSTM, and CNN-LSTM models and in the overall prediction, BPNN, CNN, LSTM, and CNN-LSTM models all have good results in the overall prediction [6]. Wu et al. proposed a wave energy prediction model using an improved BPNN model and an improved ensemble empirical mode decomposition. The wave energy is predicted by this model, and finally, the validity of the prediction model is verified by using the actual measured wind waves of the real ocean as an example [7]. Wu et al. decomposed the original wind speed sequence into a set of intrinsic mode functions using variational mode decomposition and then combined the high-performance multiview prediction and interpretable temporal dynamic insight based on the attentional deep learning model. The method is eventually used to predict wind speed from meteorological data with perfect results [8]. Lv and Wang similarly proposed an improved hybrid time series decomposition strategy for effective wind speed prediction, and the test results showed better results compared with existing decomposition strategies, optimization techniques, and deep learning predictors [9]. The use of neural network models in satellite navigation data prediction has also been investigated by several scholars. Yaqi et al. [10] predicted the broadcast ephemeris orbit error with the particle swarm optimization (PSO)-BPNN model and applied the final prediction results to error compensation. The model shows a strong fitting ability and prediction effect on the broadcast ephemeris orbit error, according to the trials. Changdong et al. [11] corrected the BDS satellite clock aberration using a neural network and improved the prediction accuracy compared with that of the traditional correction model. Bohua et al. [12] used a convolutional neural network to identify the outliers of BDS satellite clock aberration data to greatly improve the accuracy of removing the outliers of the clock aberration data. Ye et al. [13] suggested a method for predicting satellite clock aberration in the medium and long run. However, this method does not effectively address the prediction accuracy of the satellite in the short term.

Neural network models are simulated neuronal structures that are self-learning and self-adaptive and have significant effects for some nonlinear mapping relations [14, 15]. The backpropagation (BP) neural network is one of the feed-forward multilayer perceptrons, whose training process is a model that reverses the expected output and the actual output as error for data prediction by the backpropagation neural networks (BPNNs) [16]. The BP neural network is a neural network model that propagates backward according to the error value and has the advantages of a simple model, fast training, and self-adaptation compared with CNN and RNN when dealing with one-dimensional data of broadcast ephemeris error. Although the BP neural networks have a strong learning ability, the setting of initial weights affects the convergence speed and performance of the model [17, 18]. The classic particle swarm algorithm (PSO) is an intelligent optimization algorithm that simulates a flock of foraging birds by iterating the position and velocity of the particles in the swarm through the fitness function to solve for the optimal particles and eventually apply the optimized initial weight values to the BP neural network. The advantages of the PSO algorithm are its strong global search capability, simple structure, and easy implementation. However, it also has some disadvantages; for example, the setting of hyperparameters can affect the convergence results of the algorithm and it is easy to fall into local optimal solutions [19]. One of the drawbacks of the particle swarm algorithm will be addressed by intervening in the selection of hyperparameter values to make its algorithm converge faster while avoiding the emergence of local optimal solutions. This paper will make three main contributions:

- Improvement of inertia weights in the PSO algorithm. By adopting the dynamic adjustment of the inertia weights with a nonlinear function, the global optimization-seeking ability of the PSO algorithm is enhanced to avoid the emergence of local optimal solutions.
- Improvement of learning factors in the PSO algorithm. With the depth of iteration, different learning factors are selected to make the model converge faster.
- Apply the IPSO-BP model to the BDS-3 satellite broadcast ephemeris error correction. The validity of the model is verified by the latest BDS satellite broadcast ephemeris data.

## 2. Broadcast Ephemeris Data Error Analysis

### 2.1. Error Data Preprocessing

BeiDou satellite data are used as experimental data in this work. China constructed and operates the BeiDou satellite navigation system, which is a global satellite navigation system. The BeiDou system's third phase (BDS-3) was finished in 2020 and it is now capable of providing high-precision location and timing services to the world. The satellite ephemeris generally includes the broadcast ephemeris, ultrafast orbit, fast orbit, and final orbit ephemeris data. The ultrafast orbit data have a delay of 3 h and a sampling time of 15 min. The fast orbit data are delayed by 17 h and sampled for 15 min. The final orbit data are delayed for 12 d and sampled for 15 min. The final orbits can be approximated as real orbit data [20].

The BDS-3 broadcast ephemeris contains six Keplerian orbital roots and nine ingress parameters for all satellites observed by the current observatory under the toe reference ephemeris, and the satellite position and velocity at time “*t*” under the “toe” reference ephemeris can be calculated based on the formula of the broadcast ephemeris for calculating the satellite position [21].

The receiver independent exchange (RINEX) format satellite ephemeris data in this research are provided by the iGMAS Wuhan station. As real-time satellite position data, the BDS broadcast ephemeris is sampled once every hour. Under the reference of the same satellite in the same ephemeris, the BDS broadcast ephemeris orbit error can be obtained by determining the difference between the real-time position calculated by the broadcast ephemeris and the final orbit ephemeris. The calculation formula is as follows:

(1) $$\begin{array}{c} \left\{\begin{array}{c} \Delta x=x_{f}-x_{p},\\ \Delta y=y_{f}-y_{p},\\ \Delta z=z_{f}-z_{p}, \end{array}\right. \end{array}$$

where Δ*x*, Δ*y*, and Δ*z* represent the errors in the *x*-, *y*-, and *z*-directions, respectively; *x*_*f*_, *y*_*f*_, and *Z*_*f*_ represent the broadcast ephemeris under the *x*, *y*, and *z* coordinate values, respectively; and *x*_*p*_, *y*_*P*_, and *z*_*p*_ represent the values of the *x*, *y*, and *z* coordinates, respectively, in the final orbital ephemeris.

### 2.2. Error Data Analysis

**Figure 1:**
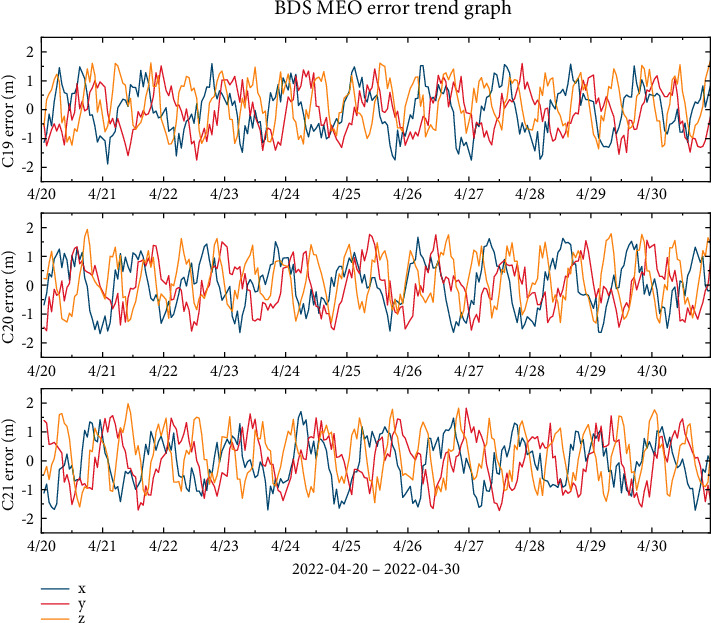
BDS-3 MEO broadcast ephemeris error trend chart.

**Figure 2:**
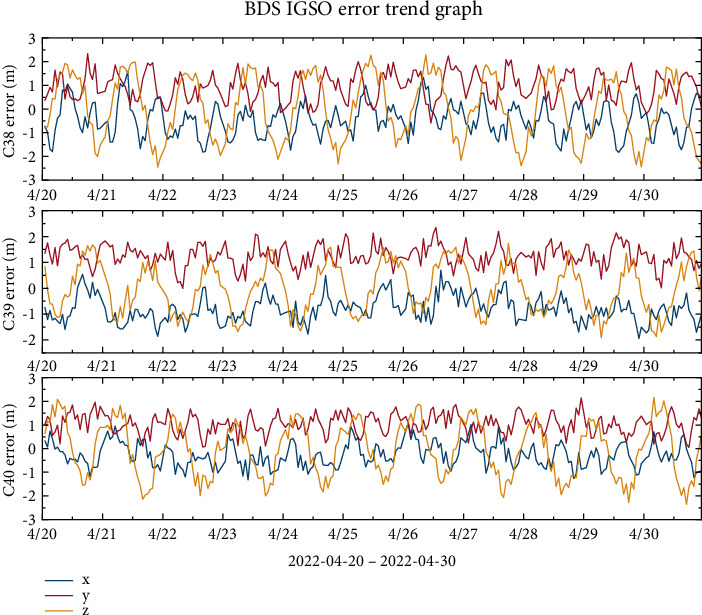
BDS-3 IGSO broadcast ephemeris error trend chart.

Without loss of generality, this work estimates the broad-cast ephemeris orbit error data for the BDS-3's IGSO and MEO satellites over a continuous period from April 20, 2022, to April 30, 2022. Among these satellites, C19, C20, and C21 are the BDS-3 MEO satellites, and C38, C39, and C40 are the BDS-3 IGSO satellites.  Figure 1 shows the orbital errors of the three MEO satellites in the *x*-, *y*-, and *z*-directions, which are all in the range of ±1.5 m.  Figure 2 shows that the orbital errors of the three IGSO satellites in the *y*-direction are between 0 and 2 m, while the errors in the *x*- and *z*-directions are in the range of ±2 m and noisy data are present. According to the trend of the error curve in the figure, it is observed that there are different oscillation patterns in each direction, but these patterns cannot be completely eliminated by the current dynamical model.

During the actual operation of satellites, they are affected by many different factors related to their natural environment, including the nonspherical gravity of the Earth, N-body uptake, solid tides, and ocean tides. [22]. The presence of these factors causes the broadcast ephemeris orbit to deviate from the actual orbit, thus producing errors. Theoretically, the accuracy of the models of these regression components should have a direct impact on the accuracy of the broadcast ephemeris orbits; however, the development of correct regression models has recently faced some difficulties.

In addition to the six Keplerian roots ($\sqrt{a}$, ⅇ, Ω_0_, *ω*, *i*_0_, and *M*_0_), the BDS-3 broadcast ephemeris orbit data contain nine ingress parameters ($\dot{I}$, $\dot{\Omega }$, Δ*n*, *C*_*us*_, *C*_*uc*_, *C*_*is*_, *C*_*ic*_, *C*_*rs*_, and *C*_*rc*_) [21], as shown in Table 1. The satellite positions and velocities are calculated from these uptake parameters. Therefore, the ingress parameters are crucial for errors in the broadcast ephemeris orbit, and they also act as influencing factors that cause the broadcast ephemeris orbit to deviate from the actual orbit. These factors are fully utilized to study the relationship between them and the broadcast ephemeris orbit error and then the nonlinear mapping relationship between them is found with the BPNN model.

## 3. Introduction to the Broadcast Ephemeris Orbit Correction Model

### 3.1. BPNN-Based Broadcast Ephemeris Orbit Correction Model

**Figure 3:**
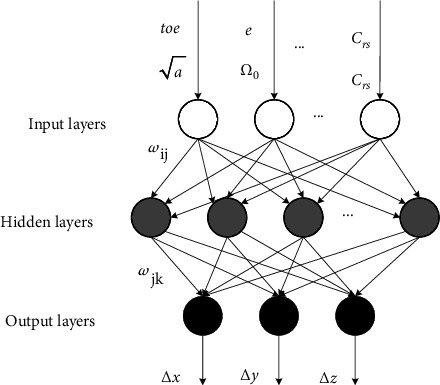
BPNN model diagram.

**Figure 4:**
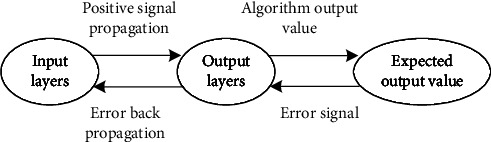
BPNN learning process.

The BPNN model is a backward-updating neural network model that is based on the difference between the actual and expected output signal values. This expected signal value is based on the principle of most rapid gradient descent. Theoretically, a three-layer BPNN can complete the mapping relationship in any dimension. In this paper, a three-layer BPNN model is used, and its structure is shown in Figure 3. The BPNN is continuously updated with the number of rounds of network training iterations, the output error of the BPNN slowly decreases and the optimal solution is finally obtained [23]. The process of reverse updating the BPNN is shown in Figure 4.

The input parameters in the BP three-layer network model are the reference moment, Keplerian orbit root number, and regenerative parameters, for a total of 16 parameters. The output represents the satellite's error in the *x*-, *y*-, and *z*-directions.

### 3.2. IPSO-BPNN Real-Time Correction Model

#### 3.2.1. Particle Swarm Optimization (PSO)

PSO is a computational intelligence method that simulates a flock of bird foraging activity by calculating the distance and speed between individual particles to optimize the population as a whole. [24]. The core algorithmic idea is that in D-dimensional space, there are *N* particles, and each of the *N* particles is searching for its individual position and group position to reach the optimum. The running speed of the particles is *V*_ⅈ,*j*_^*t*^, and each particle finds its optimum position within the group position after many iterations of each individual position and group position to constrain the movement speed. The traditional PSO algorithm is formulated as follows:

(2) $$\begin{array}{c} \begin{array}{c} x_{i,j}^{t}=\omega v_{i,j}^{t}+c_{1}r_{1}\left(p_{i,j}^{t}-x_{i,j}^{t}\right)+c_{2}r_{2}\left(p_{g,j}^{t}-x_{i,j}^{t}\right) \end{array}\text{,} \end{array}$$

(3) $$\begin{array}{c} \begin{array}{c} x_{i,j}^{t+1}=x_{i,j}^{t}+v_{i,j}^{t+1} \end{array}\text{,} \end{array}$$

where *ω* represents the inertia weight; *c*_1_ and *c*_2_ represent learning factors; *r*_1_ and *r*_2_ represent random numbers 0 and 1; and *V*_*i*,*j*_^*t*^, *x*_ⅈ,*j*_^*t*^, *p*_*g*,*j*_^*t*^ and *p*_ⅈ,*j*_^*t*^ represent the speed, location, group optimum, and individual optimum, respectively.

The traditional particle swarm algorithm faces a problem in that the inertia weights in equation (2), *c*_1_ and *c*_2_ are learning factors that can lead to instability and early convergence problems in the algorithm [25]. Therefore, in recent years, some scholars have proposed many particle swarm algorithms, such as the SPSO [26] and QPSO [27] algorithms. Among them, the SPSO algorithm changes the search space from a real-valued space to a set of selected-valued spaces, which is optimized by reducing the computational complexity of the fitness function. The QPSO algorithm optimizes the PSO algorithm by updating the particle positions according to the average optimal position of the particles. The IPSO algorithm in this paper will intervene by selecting values of hyperparameters and using nonlinear functions to dynamically adjust the values of inertia weights and learning factors to make its algorithm converge faster while avoiding the emergence of local optimal solutions. In addition, the IPSO algorithm also has the advantage of simple results and easy implementation. Therefore, it is more suitable for the optimization of the BPNN network models.

#### 3.2.2. Improvement in the Inertia Weights in PSO

At various stages of convergence, the IPSO algorithm improves the determination of inertia weight values and learning factor values. [28]. According to the experimental investigation, the value of *ω* in this algorithm has a significant impact on the overall performance of the population search; a lower *ω* value corresponds to greater ease of obtaining a local optimum, while a greater *ω* value makes it easier to achieve the global optimum. As a result, a larger *ω* value should be chosen for global optimization at the beginning of the algorithm iteration, and a lower *ω* value should be chosen for local optimization at the end of the algorithm iteration. Since the curve of the sinusoidal function in the [0, *π*] interval first rises and then falls, combined with the selection of the *ω* parameter to first use a smaller value for the motion, it gradually expands the range explored by PSO and enhances the global search capability, then continuously updates the global optimal value, and finally, reduces the inertia weight to enhance the local search capability, accelerating the convergence of PSO. The inertia weight improvement formula is as follows:

(4) $$\begin{array}{c} \begin{array}{c} \omega =\omega_{\min}+\left(\omega_{\max}-\omega_{\min}\right)\cdot \sin\left(\frac{\pi t}{T}\right) \end{array}\text{,} \end{array}$$

where *ω*_max_, *ω*_min_, and *T* are the maximum weight, minimum weight, and maximum number of iterations, respectively.

**Figure 5:**
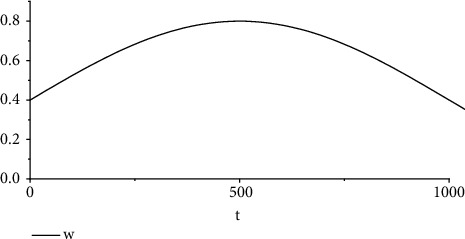
*ω* as a function of time.

For *ω*_min_ of 0.4 and *ω*_max_ of 0.8, the curve for *ω* plotted versus time is shown in Figure 5.

#### 3.2.3. Improvement in the Learning Factor in PSO

To address the drawback of the premature convergence of PSO, the calculation of the learning factor is improved; here, a larger (smaller) value of *c*_1_ makes it easier (harder) for the individual position of the particles to achieve superiority, while a larger (smaller) value of *c*_2_ makes it easier (harder) for the group position of the particles to achieve superiority. To enable the PSO algorithm to rapidly identify the global extremal solution, *c*_1_ and *c*_2_ can be adjusted as follows:

(5) $$\begin{array}{c} \left\{\begin{array}{c} c_{1}=\frac{\left(c_{s}-c_{e}\right)•\left(T-t\right)}{T}+c_{e},\\ c_{2}=4-c_{1}, \end{array}\right. \end{array}$$

where the values of *c*_*s*_ and *c*_*e*_ are between 1 and 4.

**Figure 6:**
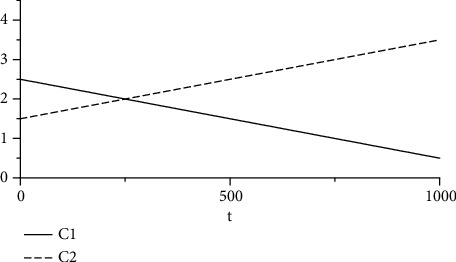
Learning factor evolution with time.

For *c*_*s*_ taken as 2.5 and *c*_*e*_ taken as 0.5, the obtained *c*_1_ and *c*_2_ are plotted versus time in Figure 6.

#### 3.2.4. Parameter Determination of the IPSO-BPNN Model

The beneficial effect of the BPNN model depends strongly on the setting of the network model parameters and is prone to suffer from drawbacks such as trapping in local optima and slow convergence. Improved particle swarm optimization, on the other hand, can optimize population data and discover the best solution in the population particles. In this paper, IPSO is used to choose all hyperparameters of the BPNN model to optimize the BPNN, prevent the model from reaching local optima, improve the BPNN's learning ability, and accelerate convergence.

*(1) BPNN Parameter Determination*. To ensure that the model can accurately map the relationship between the forecast orbital parameters and the errors of the BDS-3 broadcast ephemeris, the reference moment, the Keplerian orbital root number and regenerative parameters in the forecast orbital data are selected as the inputs of the IPSO-BPNN model, for a total of 16 parameters. The model outputs are Δ*x*, Δ*y* and Δ*z*. *m* are set to 16, *n* is set to 3, and *l* is the hidden layer.

(6) $$\begin{array}{c} \begin{array}{c} l=\sqrt{m+n}+a,a\in \left[1,10\right] \end{array}\text{.} \end{array}$$

The training objective is 0.001 and the learning rate is 0.01. The number of model iterations is 1000, the training target is 0.001, and the learning rate is 0.01.

*(2) IPSO Parameters and Fitness Function Settings.* The purpose of IPSO is to find each hyperparameter suitable for the BPNN so that the population dimension *D* is the number of all hyperparameters in the neural network; then, the algorithm randomly generates the set of all particles. *p*_*g*,*j*_^*t*^ and *p*_*i*,*j*_^*t*^ are initialized in PSO and *c*_*s*_ and *c*_*e*_ are initialized for the learning factor.

The number of example populations is 250; the values of the learning factors *c*_*s*_ and *c*_*e*_ affect the values of *c*_1_ and *c*_2_. Here, *c*_*s*_ and *c*_*e*_ are taken as 2.5 and 0.5, respectively, and the inertia weights *ω*_max_ and *ω*_min_ are taken as 0.8 and 0.4, respectively.

The fitness function can help the particle adjust the particle position and velocity, and its *mapping* relationship is an inverse proportional function, where the fitness function is given as the following equation:

(7) $$\begin{array}{c} \begin{array}{c} f=\sqrt{\frac{1}{n}\overset{n}{\underset{i=1}{\sum }}{\left[f\left(x_{i}\right)-y_{j}\right]}^{2}} \end{array}\text{,} \end{array}$$

where *n*, *f*(*x*_*i*_), and *y*_*i*_ represent the number of samples trained, the PSO predicted output value and the PSO expected output value, respectively.

#### 3.2.5. IPSO-BPNN Algorithm Flow

The BDS-3 broadcast ephemeris data in a continuous time segment are prepared, and the satellite forecast position is calculated using the formula for the satellite position and velocity. Then, the orbit error data are calculated using the satellite precision orbit data. Since the parameters of the broadcast ephemeris data are not at the same order of magnitude, it is not possible to use the neural network for training, and the data must be normalized to eliminate the differences in the magnitude. The parameters are mapped to the range between 0 and 1 using the normalization formula.

(8) $$\begin{array}{c} \begin{array}{c} y=\frac{\left(x-\bar{X}\right)}{\left(x_{\max}-x_{\min}\right)} \end{array}\text{,} \end{array}$$

where *y*, $\bar{X}$, *x*_max_, and *x*_min_ are the mapped value, data mean, maximum value in the sample, and minimum value in the sample, respectively.

**Figure 7:**
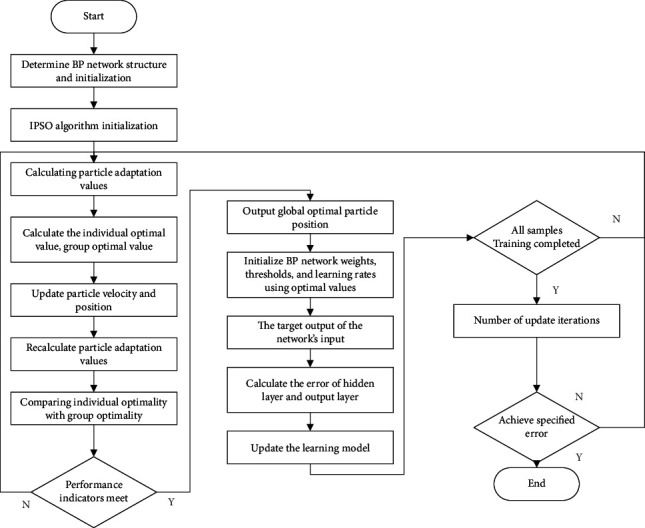
IPSO-BPNN flowchart.

Then, the output of the IPSO algorithm is utilized as the training input for the BPNN, the preprocessed data are fed into the network for model training, and the IPSO-BPNN model's error prediction value is rectified based on the original error. The detailed process of the IPSO-BPNN satellite orbit error-correction model is shown in Figure 7.

## 4. Results and Discussion

In this paper, the ephemeris data of all BDS-3 satellites are selected for a total of 22 days from April 20, 2022, to May 12, 2022. According to the broadcast ephemeris orbit data update frequency of once per hour, a total of 528 data items are calculated for a single satellite after the original data. The number of satellites used to collect the data and the number of data items are shown in Table 2. The data are divided into two parts according to time, where the data from April 20 to May 9, 2022 are used for model training and the data from May 10 to May 12 are used for model testing, and then the results of the different modified models are compared.

To ensure that the IPSO-BPNN correction model is feasible, the BPNN, PSO-BPNN, and IPSO-BPNN correction models are compared and evaluated. Meanwhile, to verify the feasibility of other models on BDS-3 satellite broadcast ephemeris data, the LSTM models are added for comparison experiments. There are four models and their experimental data are used for both BDS-3 MEO and IGSO satellite ephemeris data.

### 4.1. BDS-3 MEO Error Correction Results

**Figure 8:**
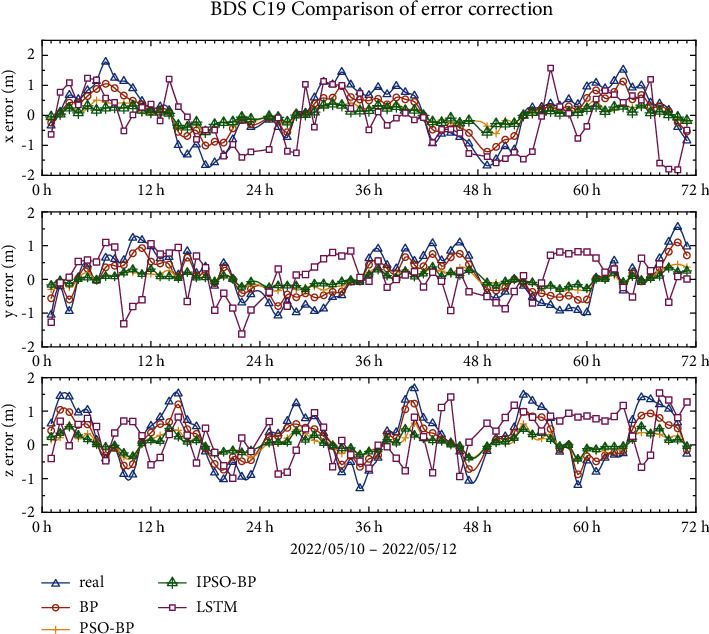
C19 broadcast ephemeris orbit error-correction curve.

**Figure 9:**
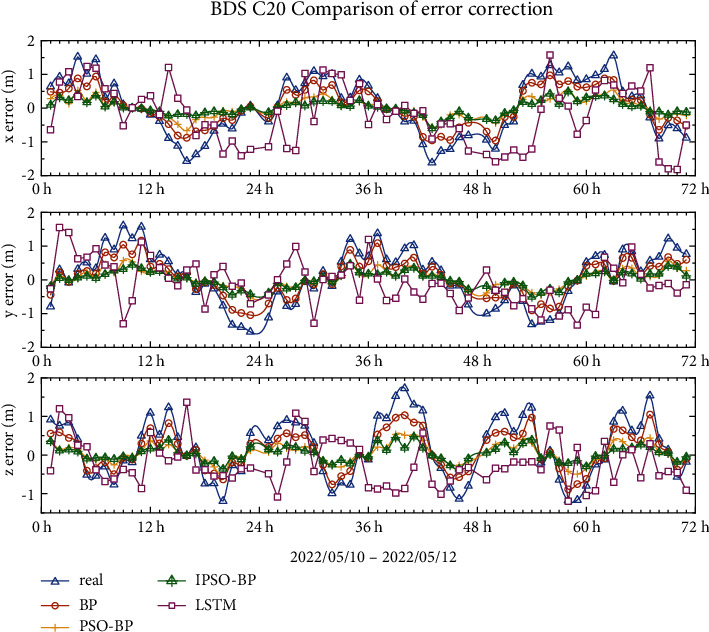
C20 broadcast ephemeris orbit error-correction curve.

The error profiles of the BDS-3 MEO satellites C19 and C20 are shown in Figures 8 and 9, with true errors of less than 2 m in all three orientations. The graph shows the change curves of the BP model, PSO-BP model, IPSO-BP model, and LSTM comparison model after the error correction within 3 days, and it can be seen from the model comparison curves that the repair effect of LSTM is more similar to the BP model but less stable than the BP model, and the repair ability of these two models is not very obvious. The results of the PSO-BP and IPSO-BP models are more similar, but the IPSO-BP model is more stable than the PSO-BP model.

Tables 3and 4 show the mean, mean squared error, and standard deviation of the BPNN, PSO-BPNN, and IPSO-BPNN models for MEO satellites in the *x*-, *y*-, and *z*-directions. The results indicate that the error repairability of the LSTM model is approximately 15%, while that of the BP model is approximately 34%. It is obvious that the BP model has better repairability than LSTM. However, in the comparison of the BP model, PSO-BP model and IPSO-BP model, the IPSO-BP model is better for broadcast ephemeris orbit error correction. The RMS error and SDT in the *x*, y, and *z* directions for the IPSO-BPNN model are decreased by 8–24 percent compared to the PSO-BPNN model.

### 4.2. BDS-3 IGSO Error Correction Results

**Figure 10:**
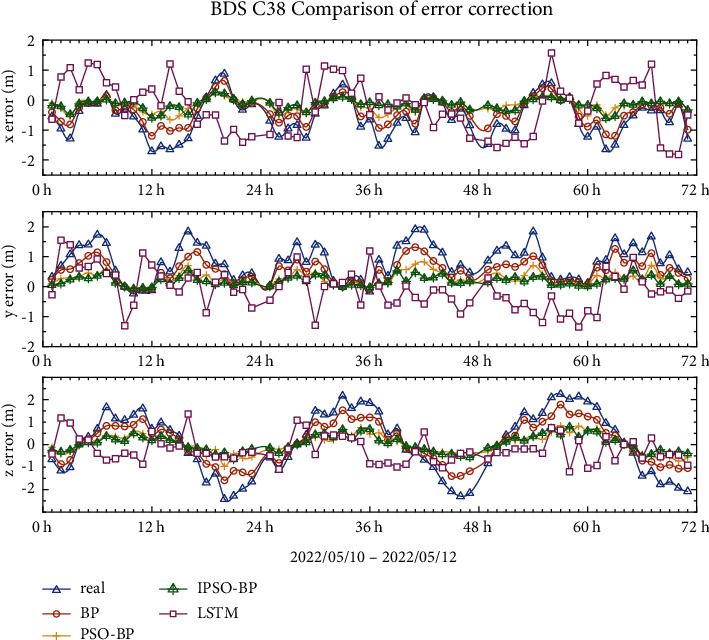
C38 broadcast ephemeris orbit error-correction curve.

**Figure 11:**
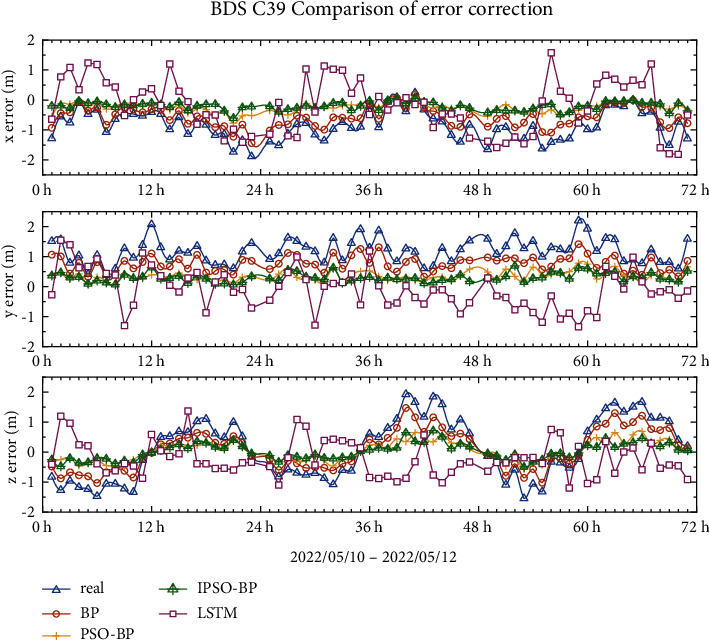
C39 broadcast ephemeris orbit error-correction curve.

Compared with those of the three generations of BDS-3 MEO satellites, the curves of the IGSO errors in the *x*-, *y*-, and *z*-directions are distinctly different, with no obvious regularity. As shown in Figures 10 and 11, the error range of C38 is between −2 m∼1 m in the *x*-direction, −0.5 m∼2 m in the *y*-direction, and −2.5 m∼2.5 m in the *z*-direction. The trend of the curves in the figure shows that the LSTM model has the worst effect among all models in terms of repair ability, while the BP model has some correction effect, but the results are not ideal. The IPSO-BP model results are better than those of the PSO-BP model and its output is closer to 0 m. The trend of the error curve in star C39 changes again, but the IPSO-BPNN model's correction performance in the *x*-, *y*-, and *z*-directions is better than that of the PSO-BPNN model.

Tables 5 and 6 demonstrate that among the three error correction models for the IGSO satellite, the IPSO-BPNN model is the most effective. This model's RMS error correction rate in the *x*-, *y*-, and *z*-directions is 62–74%. The IPSO-BPNN model's RMS and SDT in the *x*-, *y*-, and *z*-directions are lowered by 6% to 28%, respectively, compared to the PSO-BPNN model, considerably improving the accuracy of the broadcast ephemeris.

### 4.3. Error-Correction Results for All BDS-3 Satellites

To verify the feasibility of this model for other BDS-3 satellites, multiple broadcast ephemeris datasets are tested, and the corrected spatial distance values of broadcast ephemeris orbits are compared to the corresponding posterior precision ephemeris orbits. The correction rate of the mean square error of the orbit errors before and after the correction of multiple satellites is compared, and the model's feasibility is validated by comparing the correction rate of the RMS of the orbit errors before and after the correction.

The test results are calculated in the three directions after the correction with equation (9). The calculation formula is as follows:

(9) $$\begin{array}{c} \begin{array}{c} d=\sqrt{\Delta x^{2}+\Delta y^{2}+\Delta z^{2}} \end{array}\text{,} \end{array}$$

where Δ*x*, Δ*y*, and Δ*z* represent the mean squared error in the *x*-, *y*-, and *z*-directions, respectively.

The results are shown in Table 7.

Based on the mean square error correction rates of multiple BDS-3 satellites for the different correction models, compared with the BP model, the LSTM model improves the error correction rate of the broadcast ephemeris orbit for MEO satellites from 2.7% to 20.7% and the error correction rate of the broadcast ephemeris orbit for IGSO satellites from 2% to 24.5%. The model stability is poor.

In the BP model, PSO-BP model, and IPSO-BP model comparison tests, the IPSO-BPNN model improves the broadcast ephemeris orbit error correction rate of the MEO satellites in the range of 71.1–84%, and the broadcast ephemeris orbit error correction rate of IGSO satellites is improved by 70.2–80.2%, indicating that the IPSO-BPNN model has a better correction effect on satellites with different orbit types. Among the 23 MEO satellites, 12 satellites show the mean square error correction rates above 80% and the highest rate is above 84%. In this experiment, we do not exclude the existence of satellite noise data that interfere with the experiment and cause fluctuations in the accuracy, but the IPSO-BPNN broadcast ephemeris orbit correction model is more accurate than the BPNN and PSO-BPNN models overall. The correction rate is improved by 55.6–74.8% compared with the BPNN model and by 14.2–56.8% compared with the PSO-BPNN model. These studies showed that the optimization of the algorithm in this paper clearly produces improved results.

## 5. Conclusion

There is a certain error between the broadcast ephemeris orbit and the precise orbit of the satellite navigation system, and this error cannot be completely eliminated by the existing dynamical model. Through error analysis, it is found that there are some periodic regular phenomena in the broadcast ephemeris orbit errors. In this paper, the orbit error is modeled for this phenomenon using an improved particle swarm algorithm to optimize the BP neural network. Model improvement mainly uses nonlinear functions for inertia weights and learning factors to dynamically adjust the algorithm to accelerate its convergence while avoiding trapping in local optimal solutions. Experiments using BDS-3 satellite data show that the proposed method can construct an accurate orbit error model, and the accuracy of broadcast ephemeris orbit is significantly improved compared with BPNN and PSO-BPNN. In future research work, other deep learning methods, such as GRU and GAN, will be further combined with satellite navigation data processing to verify their effects on data accuracy improvement.

## Figures and Tables

**Table 1:** Broadcast ephemeris camera parameters description table.

Parameters	Description	Unit
$\dot{I}$	Rate of change in orbital inclination	rad/s
$\dot{\Omega }$	Rate of change in the equatorial diameter of the ascending intersection	rad/s
Δ*n*	Correction value of the angular velocity at the level of proximity	rad/s
*C*_*us*_, *C*_*uc*_	Amplitudes corrected by the sine and cosine summation terms of the latitude amplitude angles	rad
*C*_*is*_, *C*_*ic*_	Amplitude of the correction of the sine and cosine summation terms of the orbital inclination angle	rad
*C*_*rs*_,*C*_*rc*_	Amplitude of correction of the sine and cosine summation terms of the orbital radius	m

**Table 2:** Datasets description table.

Parameters	Broadcast ephemeris data	Precision ephemeris data
PRN	BDS-3 (C19–C45)	BDS-3(C19–C45)
Date	2022-04-20 01 : 00∼2022-05-12 23 : 00	2022-04-20 01 : 00∼2022-05-12 23 : 00
File type	xxxx.rnx.gz	xxxx.SP3.gz
Lines per file	Approximately 135000	Approximately 11650
File size	Approximately 10000 KB	Approximately 700 KB
Number of files	22	22
Parsing format	RINEX format	SP3 format

**Table 3:** C19 broadcast ephemeris orbit error-correction table.

Model	*x*			*y*			*z*		
	Mean/m	STD/m	RMS/m	Mean/m	STD/m	RMS/m	Mean/m	STD/m	RMS/m
Real	0.729	0.875	0.869	0.597	0.698	0.693	0.681	0.802	0.796
BPNN	0.467	0.564	0.560	0.390	0.458	0.454	0.447	0.531	0.527
PSO-BPNN	0.220	0.270	0.268	0.176	0.209	0.208	0.198	0.246	0.244
IPSO-BPNN	0.184	0.225	0.223	0.137	0.167	0.166	0.176	0.223	0.222
LSTM	0.749	0.891	0.885	0.528	0.666	0.661	0.475	0.593	0.588

**Table 4:** C20 broadcast ephemeris orbit error-correction table.

Model	*x*			*y*			*z*		
	Mean/m	STD/m	RMS/m	Mean/m	STD/m	RMS/m	Mean/m	STD/m	RMS/m
Real	0.735	0.854	0.848	0.671	0.799	0.793	0.674	0.778	0.772
BPNN	0.486	0.564	0.560	0.445	0.536	0.532	0.429	0.499	0.496
PSO-BPNN	0.228	0.279	0.277	0.205	0.254	0.252	0.200	0.247	0.245
IPSO-BPNN	0.172	0.215	0.213	0.187	0.232	0.230	0.158	0.195	0.193
LSTM	0.649	0.782	0.777	0.537	0.650	0.645	0.571	0.669	0.664

**Table 5:** C38 broadcast ephemeris orbit error-correction table.

Model	*x*			*y*			*z*		
	Mean/m	STD/m	RMS/m	Mean/m	STD/m	RMS/m	Mean/m	STD/m	RMS/m
Real	0.519	0.627	0.623	0.480	0.572	0.567	1.206	1.390	1.380
BPNN	0.346	0.423	0.420	0.304	0.371	0.368	0.777	0.890	0.884
PSO-BPNN	0.164	0.202	0.200	0.168	0.210	0.209	0.347	0.414	0.411
IPSO-BPNN	0.139	0.182	0.180	0.126	0.151	0.150	0.304	0.361	0.358
LSTM	0.510	0.628	0.624	0.333	0.421	0.418	1.179	1.380	1.370

**Table 6:** C39 broadcast ephemeris orbit error-correction table.

Model	*x*			*y*			*z*		
	Mean/m	STD/m	RMS/m	Mean/m	STD/m	RMS/m	Mean/m	STD/m	RMS/m
Real	0.385	0.473	0.470	0.316	0.396	0.393	0.881	0.993	0.986
BPNN	0.262	0.328	0.326	0.215	0.258	0.256	0.568	0.653	0.648
PSO-BPNN	0.131	0.165	0.164	0.125	0.160	0.159	0.283	0.329	0.327
IPSO-BPNN	0.109	0.135	0.134	0.116	0.149	0.148	0.226	0.271	0.269
LSTM	0.390	0.489	0.486	0.284	0.353	0.350	0.623	0.780	0.774

**Table 7:** Broadcast ephemeris orbit error-correction table.

Satellite type	PRN	RMS/m					Improvement rate			
		Real	LSTM	BPNN	PSO-BPNN	IPSO-BPNN	LSTM	BPNN	PSO-BPNN	IPSO-BPNN
MEO	C19	0.791	0.658	0.518	0.242	0.205	16.90%	34.6%	69.5%	74.1%
	C20	0.810	0.682	0.533	0.260	0.214	15.80%	34.2%	67.9%	73.6%
	C21	0.818	0.705	0.531	0.259	0.145	13.80%	35.1%	68.3%	82.3%
	C22	0.814	0.659	0.534	0.246	0.156	19.00%	34.3%	69.7%	80.8%
	C23	0.854	0.718	0.560	0.258	0.137	16.00%	34.5%	69.8%	84.0%
	C24	0.849	0.706	0.562	0.277	0.143	16.80%	33.8%	67.3%	83.2%
	C25	0.737	0.63	0.480	0.322	0.213	14.50%	34.9%	56.3%	71.1%
	C26	0.714	0.695	0.467	0.221	0.119	2.70%	34.6%	69.0%	83.4%
	C27	0.722	0.639	0.467	0.224	0.161	11.50%	35.3%	68.9%	77.7%
	C28	0.724	0.63	0.473	0.341	0.123	12.90%	34.7%	52.9%	83.1%
	C29	0.723	0.616	0.465	0.339	0.198	14.80%	35.7%	53.1%	72.6%
	C30	0.674	0.663	0.438	0.211	0.181	1.60%	35.0%	68.7%	73.1%
	C32	0.797	0.737	0.515	0.301	0.130	7.60%	35.3%	62.2%	83.7%
	C33	0.809	0.641	0.527	0.246	0.145	20.70%	34.8%	69.6%	82.1%
	C34	0.702	0.642	0.458	0.422	0.120	8.50%	34.8%	39.9%	82.9%
	C35	0.718	0.574	0.466	0.222	0.151	20.00%	35.0%	69.1%	79.0%
	C36	0.744	0.641	0.484	0.367	0.198	13.80%	34.9%	50.6%	73.4%
	C37	0.773	0.701	0.516	0.246	0.135	9.30%	33.2%	68.3%	82.5%
	C41	0.594	0.525	0.386	0.184	0.106	11.60%	35.0%	69.1%	82.1%
	C42	0.602	0.485	0.394	0.221	0.152	19.40%	34.5%	63.3%	74.7%
	C43	0.673	0.596	0.436	0.212	0.118	11.50%	35.2%	68.6%	82.4%
	C44	0.729	0.625	0.480	0.321	0.172	14.30%	34.1%	56.0%	76.4%
	C45	0.773	0.694	0.507	0.239	0.198	10.30%	34.4%	69.1%	74.4%
IGSO	C38	0.863	0.814	0.555	0.281	0.121	5.60%	35.7%	67.4%	86.0%
	C39	0.621	0.469	0.422	0.211	0.123	24.50%	32.0%	65.9%	80.2%
	C40	0.618	0.606	0.413	0.209	0.122	2.00%	33.1%	66.1%	80.2%

## Data Availability

The satellite ephemeris data in this paper are provided by the iGMAS Wuhan station. The data can be found at the links below: (1) https://ftp://igs.gnsswhu.cn/pub/gps/data/daily/2022/. (2) https://ftp://igs.gnsswhu.cn/pub/gps/products/mgex/.
